# Aptamer‐Mediated Reversible Transactivation of Gene Expression by Light

**DOI:** 10.1002/anie.202009240

**Published:** 2020-10-02

**Authors:** Christian Renzl, Ankana Kakoti, Günter Mayer

**Affiliations:** ^1^ LIMES University of Bonn Gerhard-Domagk-Str. 1 53121 Bonn Germany; ^2^ Center of Aptamer Research & Development University of Bonn Gerhard-Domagk-Str. 1 53121 Bonn Germany

**Keywords:** aptamers, gene expression, synthetic biology

## Abstract

The investigation and manipulation of cellular processes with subcellular resolution requires non‐invasive tools with spatiotemporal precision and reversibility. Building on the interaction of the photoreceptor PAL with an RNA aptamer, we describe a variation of the CRISPR/dCAS9 system for light‐controlled activation of gene expression. This platform significantly reduces the coding space required for genetic manipulation and provides a strong on‐switch with almost no residual activity in the dark. It adds to the current set of modular building blocks for synthetic biological circuit design and is broadly applicable.

## Graphical abstract

A three‐component light switch for programmable gene activation is reported. A photoreceptor aptamer pair facilitates reversible control of gene expression and broadens the optoribogenetic toolbox.

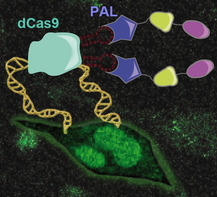

Clustered regularly interspaced short palindromic repeats (CRISPR) loci and the CRISPR‐associated (cas) genes encode for an RNA‐guided adaptive immune system in prokaryotes.^[1]^ The Cas9 protein, which is used as a programmable nuclease, specifically binds and cleaves selected DNA sequences and became a powerful tool for biotechnological research.^[2]^ Because of the easy implementation, CRISPR has become a standard in genome editing, gene regulation or DNA imaging.^[3]^ The CRISPR/Cas systems can be subdivided into three main types (Type I, II, and III) that differ in their corresponding Cas proteins.^[4]^ Among these, the Type II Cas9 protein from S. pyogenes is the best studied.^[5]^ The nuclease deficient mutant of Cas9, dubbed dCas9, enables CRISPR/dCas9 to be used as a biological navigation system, which can be functionally extended by generating effector‐dCas9 fusion proteins. Thereby, new functions such as transcriptional activation, DNA tagging, chromatin purification, RNA recruitment, DNA looping, and chromatin modification are accessible. Transcriptional activation, named CRISPR activation (CRISPRa), is achieved by fusing transactivation domains, e.g., VP64, p65, HSF1, and rta to dCas9[^3e^, ^6^] and optogenetic control is attained using CIB1 and CRY2 as photodimerizable components fused to transactivation and effector proteins.^[7]^ Optogenetic dCas9‐based activation systems involve a cluster of proteins, that overstrain the capacity of single viral payload in regard of coding space. CRY2/CIB1‐based methods, in turn, are limited by CRY2 homodimerization in light^[8]^ and split dCas9 variants are associated with higher background activity and slow off‐rates.^[7a]^ In our approach, we constructed sgRNAs having short RNA aptamer domains binding to the protein PAL.^[9]^ PAL is a LOV domain photoreceptor and interacts reversibly with the aptamer 53.^[9]^ In previous work, we embedded this aptamer into the 5′‐untranslated region of mRNA molecules, by which a light‐dependent off‐switch of gene expression was generated.^[9]^ We now sought to create on‐switches based on the PAL:aptamer system that enable the activation of gene expression upon irradiation rather than inhibiting. To this end, we created an RNA‐protein interface that reversibly activates gene expression in response to blue light by fusing PAL to the activator domains p65 and HSF1 (p65‐HSF1‐PAL, dubbed PHP). Irradiation thus induces recruitment of PHP to sgRNA tagged loci triggering transcription and gene expression (Figure 1 a).

**Figure 1:**
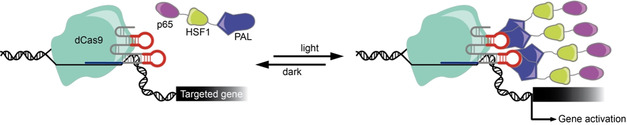
Light‐dependent reversible control of transcription activation based on the PAL‐aptamer system. Schematic of gene photoactivation by conditional recruitment of transcriptional activators to the target gene promoter. The coding space of PHP is 2109 bases.

We introduced aptamer 53 into the tetraloop (TL) and stem‐loop 2 (SL2) of sgRNAs with different stem lengths (SG6, SG9, and SG12) to allow variable positioning of PHP for transcription activation (Figure 2 a, Figure S1a–c, Supporting Information)^[6c]^ and to avoid negative impact on PAL binding in light by putative steric hindrance of the sgRNA/dCas9 complex. We first tested the capability of the modified sgRNAs to mediate Cas9‐dependent cleavage of target DNA in vitro and found explicit cleavage activity for sgRNAs SG6 and SG9 in comparison with the native, that is, non‐modified sgRNA (control) and sgRNA 2.0,^[6c]^ when a targeting seed sequence was used (Figure S1d, Supporting Information). Cleavage efficiency mediated by SG12 was diminished (Figure S2a, Supporting Information) when a targeting seed sequence was used. sgRNAs programmed with a non‐binding^[10]^ seed sequence showed no or strongly reduced activity. We next investigated transactivation of gene expression in mammalian cells. Nuclear expression of PHP in HeLa cells and the switching activity of its PAL domain was confirmed by fluorescence microscopy (Figure S2b,c and Note S2, Supporting Information). In initial experiments, an eBFP reporter was used containing eight sgRNA binding sites upstream of a minimal CMV promoter (Figure 2 a). We transiently co‐expressed dCas9, the eBFP reporter, PHP and sgRNA SG6 in HeLa cells. As control, we included the SAM system using MS2‐p65‐HSF1 (MPH) and its corresponding sgRNA 2.0 but using dCas9 without VP64.^[6c]^ Light‐dependent and aptamer specific activation of gene expression was found for sgRNA SG6 when co‐expressed with PHP, whereas no eBFP was detected in darkness (Figure S2d,e, Supporting Information). Activation of gene expression was also observed using MPH and sgRNA 2.0, which is independent of the irradiation status (Figure S2d, Supporting Information). Expression of MPH and SG6 or PHP and sgRNA 2.0 induced gene expression neither in darkness nor in light (Figure S2d, Supporting Information). We optimized the ratio of the plasmids used for transfection (Figure S2f, Supporting Information) and applied these to sgRNAs SG9 and SG12. Compared to SG6, a stronger induction of gene expression was found when using sgRNAs with stem lengths of 9 or 12 nts (Figure 2 b, Figure S1 and S2g,h, Supporting Information). No activation of gene expression was observed in darkness or when PAL‐binding incompetent point mutants of the aptamers were used, having a point mutation in the aptamer domain embedded in TL and in SL2 (SG6M21, SG9M21, or SG12M21) (Figure 2 b, Figure S1 and S2g,h, Supporting Information). We analyzed the tunability of the system, that is, dependency of gene expression on light intensity using SG9. Increasing light intensity results in an enhanced eBFP expression and a maximum at a value of 100 μW cm^−2^ (Figure 2 c). The activation efficacy was also found to be slightly stronger when using PHP compared to a variant with an altered order of the effector domains (HSF1‐p65‐PAL, HPP) (Figure 2 d). Of note, mutation of only one of the two aptamer domains (SG9M21TL, only mutated in the aptamer domain of the tetraloop; SG9M21SL2, only mutated in the aptamer domain of stem‐loop 2) in the sgRNAs leads to a strong reduction in activity (Figure 2 e, Figure S1, Supporting Information), indicating that both aptamer domains are necessary to mediate the transactivation of gene expression. This superiority of two aptamers over one was also found when using the MS2 system.^[6c]^ This observation might be explained by synergistic effects leading to higher local concentrations of transactivation domains upstream of the promoter. Furthermore, cooperative effects in PHP‐aptamer binding, mediated by dimer aptamer arrangements in the respective sgRNAs might support the recruitment and positioning of the transactivation domain. Similar results were observed when using *Metridia* luciferase as reporter gene (Figure 2 f, Figure S2i, Supporting Information), although total expression levels were lower compared to eBFP (Figure S2g, Supporting Information). *Metridia* luciferase is secreted into the cells’ supernatant and can therefore be utilized to investigate the reversibility of the PHP system. We first tested *Metridia* luciferase activity with increasing incubation times for an optimal signal‐to‐noise ratio (Figure S2j, Supporting Information). Luciferase activity was then measured 12 h after transfection and irradiation (Figure 2 g), after which the cells were kept in darkness for further 12 h. Reporter levels were found to be reduced by 50 % and could be fully restored by irradiation (Figure 2 g). Given the average half‐life of mRNAs in human cells of approx. 10 h,^[11]^ this result demonstrates reversibility of the PAL‐driven gene expression by light.

**Figure 2:**
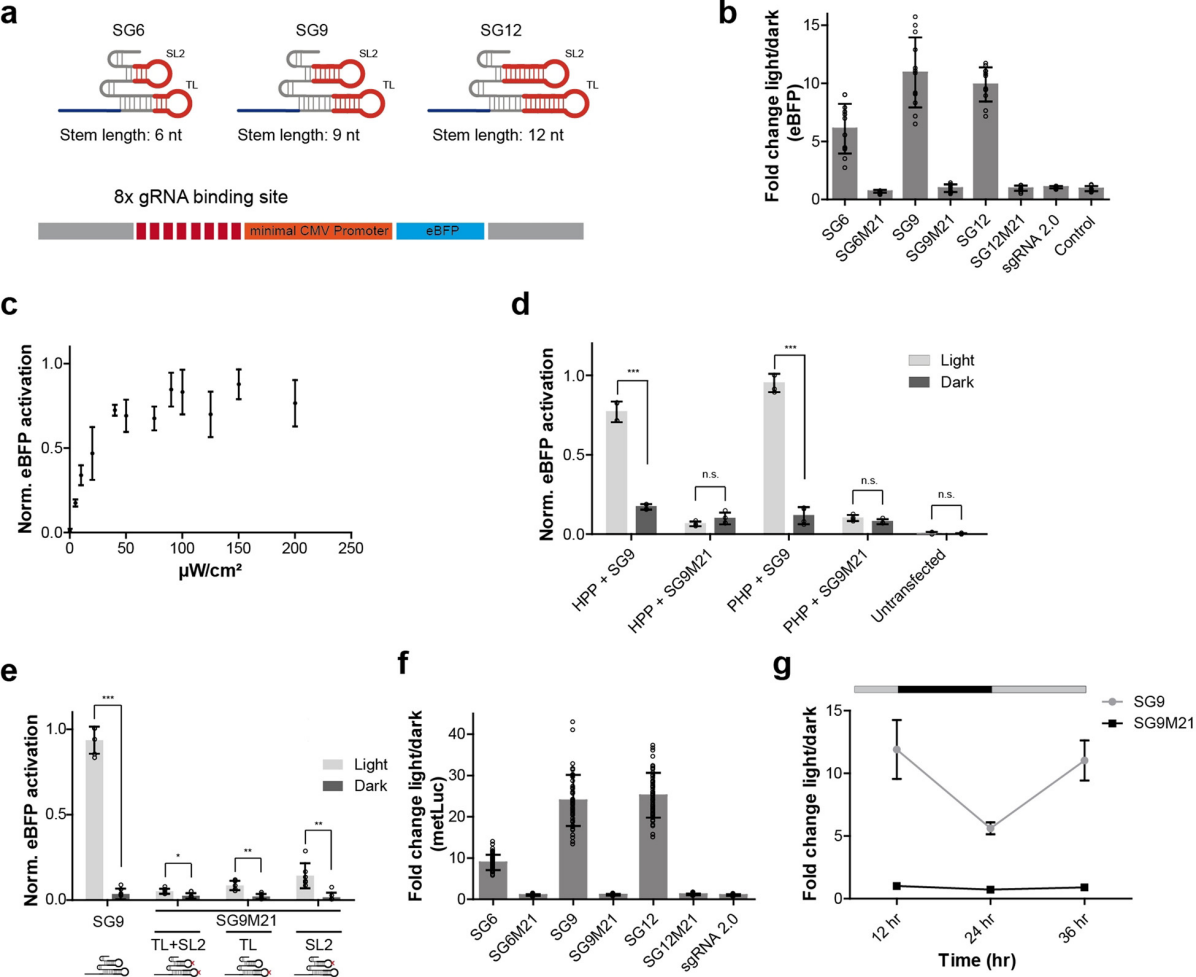
Reporter‐based activation assays. a) Schematic of PAL aptamer extended sgRNAs (top) and schematic of the promoter region of the eBFP reporter gene with sgRNA binding sites (bottom). b) Light‐dependent regulation of eBFP reporter using PAL aptamer extended sgRNAs with varying stem‐lengths (SG6, SG9 and SG12) or PAL‐binding deficient mutants (SG6M21, SG9M21 and SG12M21). c) Impact of increasing light intensity on eBFP expression using SG9. Given are mean values ± s.d normalized to the highest value using min‐max scaling (*n*=4 from 2 individual experiments). d) Influence of the arrangement of the activator domains on light‐dependent eBFP expression (PHP: p65‐HSF1‐PAL; HPP: HSF1‐p65‐PAL). The data is presented as mean values ± s.d (*n*=4 from 2 individual experiments). Welch's two‐tailed *t*‐test was performed for the fold induction in the light versus the sample in the dark. The resulting *p*‐values of the samples are given in the Supporting Information in Table S3. *p<0.05, **p<0.01, ***p<0.005 versus the sample in the dark. e) Light‐dependent induction of eBFP expression using sgRNAs having one (SG9M21 TL; SG9M21SL) or both (SG9M21TL+SL) aptamer domains, as presented in (a), mutated. Mutations are indicated in the RNA schemes below the *x*‐axis by a red *x*. Welch's two‐tailed *t*‐test was performed for the fold induction in the light versus the sample in the dark. The resulting *p*‐values of the samples are given in the Supporting Information in Table S3. *p<0.05, **p<0.01, ***p<0.005 versus the sample in the dark. f) Light‐dependent regulation of *Metridia* Luciferase expression using sgRNAs with varying aptamer stem‐lengths (SG6, SG9 and SG12) or PAL‐binding deficient mutants (SG6M21, SG9M21 and SG12M21). g) Reversible expression of *Metridia* Luciferase using SG9 or SG9M21. The bar indicates the irradiation status over time (grey: light; black: dark). Welch's two‐tailed *t*‐test was performed for the fold induction in the light versus the sample in the dark. The resulting *p*‐values of the samples are given in the Supporting Information in Table S3. *p<0.05, **p<0.01, ***p<0.005 versus the sample in the dark. The data in (b)–(g) are presented as mean values ± s.d. The data in (c) is normalized to the highest value using min‐max scaling and the data in (d) is normalized to the value obtained from PHP+SG9 in light using min‐max scaling. The data in (e) are normalized to SG9 in light using min‐max scaling (b, e, and f: *n*=6 from 3 individual experiments; c, d and g: *n*=4 from 2 individual experiments).

We next applied the PHP system to control the expression of an endogenous gene, exemplified by human achaete‐scute homolog 1 (ASCL1). ASCL1 encodes a transcription factor, which is involved in neuronal differentiation. In HeLa cells the expression of ASCL1 is low, making it a suitable target gene for artificial upregulation leading to high dynamic ranges.[^6c^, ^7a^] Initially, we used sgRNAs with a 9nt stem and targeting six different loci upstream of the human ASCL1 promoter (SG9#1 to SG9#6, Figure 3 a, Table S1, Supporting Information). For comparison, we adapted the ASCL1 loci from Nihongaki et al.,^[7a]^ showing transcriptional activation of ASCL1 regulation using the CPTS2.0 system. Among the adapted sgRNAs, those targeting positions #2 and #4 showed the strongest activation of ASCL1 expression in light using PHP (Figure 3 b). While loci #4 was also found to be effective in mediating transcriptional activation using the CPTS2.0 system, loci #2 was more potent when the SAM and split‐CPTS2.0 systems were used.^[7a]^ These findings indicate, that the combination of the effector system and loci is critical for achieving potent transcriptional activation. SG9#2 and SG9#4 revealed fold changes (light vs. dark) of 99 and 21, respectively (Figure 3 c). These values were further increased using both sgRNAs simultaneously (546, Figure 3 c). In our hands, the PHP:SG9#2 combination was found to be as potent as the MPH:sgRNA2.0 combination in gene activation, whereas the latter being light independent (Figure 3 c). The highest induction of transcription is often achieved, when sgRNAs are positioned between −25 and −550 bp upstream of the promoter,^[12]^ from which the highest activity is most likely between −25 and −200 bp.^[6c]^ The sgRNAs SG9#2 and SG9#4 are located to this region. The less effective loci might be affected by an unfavorable orientation on the DNA towards the promoter or by nucleosomes that impact the dCas9‐complex from binding to the DNA.^[13]^

**Figure 3:**
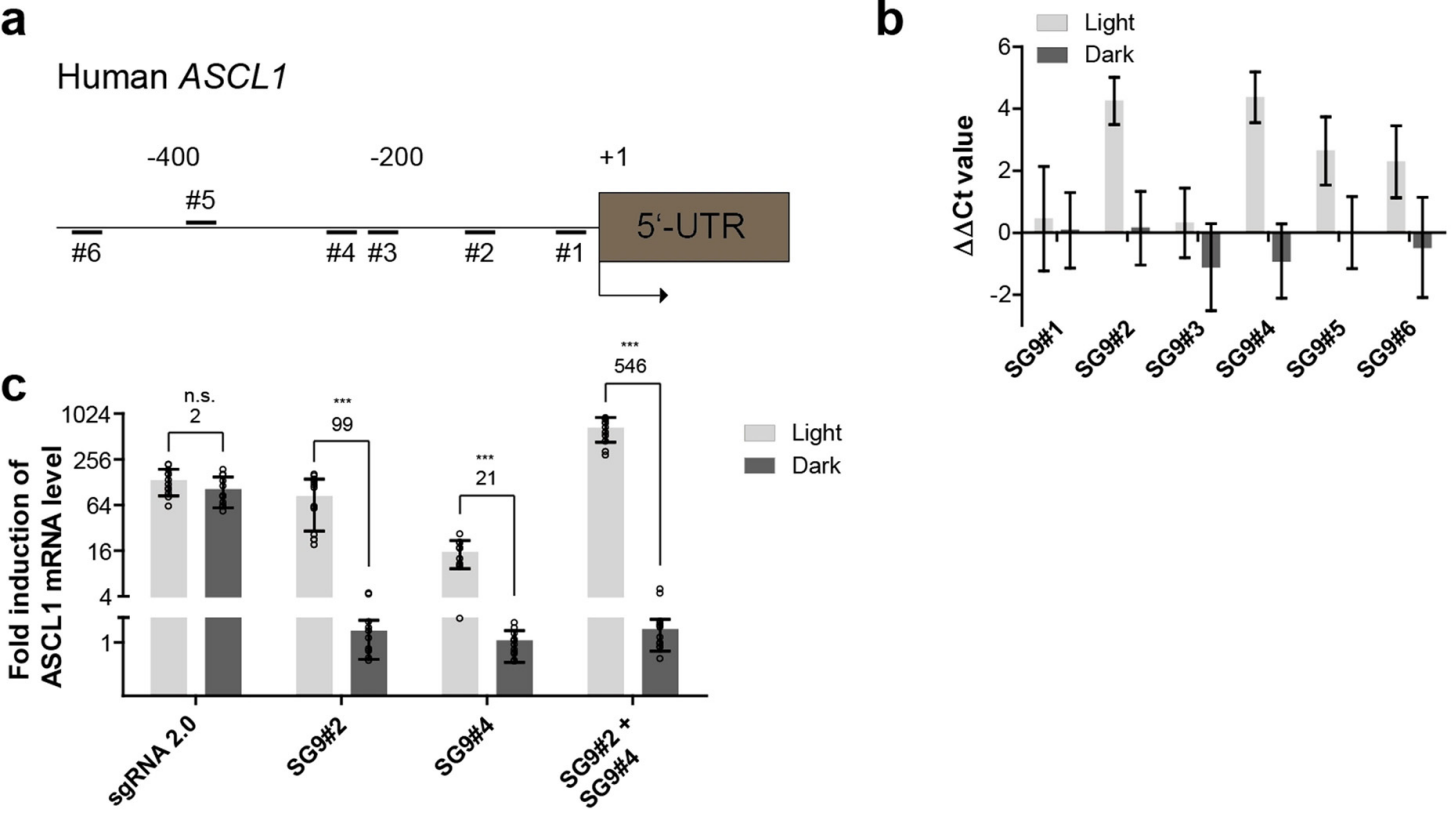
Light‐dependent activation of endogenous hASCL1. a) Schematic of the human endogenous ASCL1 promoter and spatial arrangement of utilized sgRNA binding sites. b) Light‐dependent regulation of hASCL1 expression using sgRNAs targeting varying sgRNA binding sites (Δct values were normalized to control). c) Light‐dependent regulation of endogenous hASCL1 using sgRNAs SG9#2, SG9#4 and sgRNA2.0 (Δct‐values were normalized to control sgRNA). For the fold induction of mRNA, 2 ΔΔct was calculated. Welch's two‐tailed *t*‐test was performed for the fold induction in the light versus the sample in the dark. The resulting *p*‐values of the samples are given in the Supporting Information in Table S3. *p<0.05, **p<0.01, ***p<0.005 versus the sample in the dark. Values over the bar groups represent fold‐change (light over dark) of mRNA induction measured by qPCR. The data in (b) and (c) are presented as mean values ± s.d (for b *n*=4 from 2 individual experiments with two cell culture replicates and (c) *n*=6 from 3 individual experiments with two cell culture replicates).

In conclusion, we report on an optoribogenetic method that enables the reversible activation of gene expression with blue light. The described method makes use of a light‐dependent RNA‐protein interaction and reduces the number of components of CRISPR/dCas9^[7a]^ approaches to three, that is, sgRNAs, dCas9 and PHP. The coding space of the CPTS2.0 system^[7a]^ is 4254 bases, whereas PHP requires only 50 % of that coding space (i.e. 2109 bases). This compact system needs less coding space, which might allow the integration of all components in a single virus. As this option is not yet available from other systems, stable CRISPR cell lines can be generated to be transduced with corresponding sgRNAs.[^6c^, ^11^] For example, cells or organisms that stably express dCas9 might be used, further underscoring the compactness of the light‐dependent PHP effector system. Moreover, the PHP system is not affected by homodimerization and background activity. We show, that different stem lengths influence the activity of sgRNAs, whereas results found in vitro not strictly reflect the situation observed from experiments in cells. This finding might be due to differences in intracellular environment that impact RNA performance, e.g., molecular crowding and ion composition.^[15]^ We anticipate our approach being broadly applicable in cells and in vivo, as well as for synthetic biology approaches, e.g., as building block in a multi‐component activation system that combines several aptamers^[16]^ and thereby extends the complexity of available synthetic gene circuits.

## Conflict of interest

The authors declare no conflict of interest.

## Supporting information

As a service to our authors and readers, this journal provides supporting information supplied by the authors. Such materials are peer reviewed and may be re‐organized for online delivery, but are not copy‐edited or typeset. Technical support issues arising from supporting information (other than missing files) should be addressed to the authors.

Supplementary
