# Supplementary material for: Aptamer‐Mediated Reversible Transactivation of Gene Expression by Light
Source: Angew Chem Int Ed Engl. 2020 Oct 2;59(50):22414–8. doi: 10.1002/anie.202009240 (PMC7756287; doi:10.1002/anie.202009240)
Supplement: Supplementary file 1 — Supplementary [file ANIE-59-22414-s001.pdf]

Angewandte Chemie
Eine Zeitschrift der Gesellschaft Deutscher Chemiker

Supporting Information

Aptamer-Mediated Reversible Transactivation of Gene Expression by Light

*Christian Renzl, Ankana Kakoti, and Günter Mayer**

anie_202009240_sm_miscellaneous_information.pdf

Experimental Section

Sequences: DNA sequences of the primers for amplification were ordered from Ella Biotech (Martinsried, Germany) and are provided together with the RNA sequences of the sgRNAs in the Supplementary Information.

Cell culture: For all experiments, HeLa cells (CLS, Eppenheim, Germany) were used. The cells were cultured in DMEM (Gibco Thermo Fisher Scientific, Waltham, USA) supplied with 10% FCS, 1% non-essential amino acids and 1 mM sodium pyruvate. Usually, the cells were cultivated in T-75 flasks and passaged 2-3 times weekly.

Transfection: For optogenetic gene activation experiments, 70.000 HeLa cells were seeded into 24-well plates one day before transfection. After 24h, the transfection reagents were prepared. For the first experiments with fixed nanograms of plasmid, 200 ng of reporter plasmid, 300 ng of dCas9 plasmid, 200 ng of effector plasmid and 100 ng of sgRNA plasmid were used (Figure S2d, Supporting Information). After the transfection optimization (Figure S2f, Supporting Information), fixed molar ratios were used. For each transfection a total amount of 200 fmol plasmids were added into a reaction tube and filled with OptiMEM (Gibco Thermo Fisher Scientific, Waltham, USA) to a final volume of 25 µl per transfection reaction. In a second tube 25 µl OptiMEM was mixed with 1,5 µl Lipofectamine® 2000 Reagent (Gibco Thermo Fisher Scientific, Waltham, USA). Both tubes were vortexed shortly and incubated for 5 min at 20 °C. Subsequently, the plasmid mix was added to the Lipofectamine mix. The resulting composition was vortexed shortly and incubated for 30 min at 20 °C. In parallel, the medium of the seeded cells was removed and replaced by 500 µl prewarmed OptiMEM. After the 30 min incubation time, the resulting 50 µl total transfection mix was added to the cells dropwise. The transfection was incubated for 4 hours at 37 °C and 5% CO_2. After 4 hours, the transfection mix was removed and replaced by 500 µl of DMEM + 10 % FCS. For confocal microscopy experiments 50.000 HeLa cells were seeded in black 24-well plates with clear bottom (µ-plate, ibidi, Martinsried, Germany). On the next day the cells were transfected using 500 ng of plasmid as previously described.

Confocal microscopy experiments: For the analysis of switching of PAL, HeLa cells were seeded and transfected with PHP (Supplementary Figure 2 c). On day after transfection, the cells were analyzed, using a Zeiss LSM 710 Axio Observer (Zeiss, Oberkochen, Germany) and a 40x magnification. The switching program used, first takes images in the mCherry channel (ex. 588 nm, em. 610 nm) and irradiates the cells with a laser at 405 nm for 1 min to induce the conformational change of PAL. It uses the emission filter of the eGFP channel (em. 510 nm) to select for the PAL dark fluorescence and subsequently takes images for mCherry and PAL dark fluorescence again. Pictures were taken for each channel separately and merged.

Reporter constructions: Cloning of pGL3-8xsgRNA-eBFP and pGL3-8xsgRNA-metLuc. The template for the final plasmid is the pGL3-Basic-8x-gRNA-eGFP. pGL3-Basic-8x-gRNA-eGFP was a gift from Charles Gersbach (Addgene plasmid # 60718). The backbone was amplified by PCR, excluding the eGFP sequence. The eBFP insert was PCR amplified from pU6-(BbsI)Cbh-Cas9-T2A-BFP. pU6-(BbsI)CBh-Cas9-T2A-BFP was a gift from Ralf Kuehn (Addgene plasmid # 64323). The *Metridia* luciferase insert was PCR amplified from pMetLuc2-Reporter (Clontech Takara, Saint-Germain-en-Laye, France). For the plasmid amplification the Phusion Flash High-Fidelity PCR Master Mix (Thermo Fisher Scientific) was used. The primers for the insert were designed to have an overlap with the target backbone. For the cloning, In-Fusion® HD Cloning Kit (Clontech Takara, Saint-Germain-en-Laye, France) was used according to the manufacturers protocol.

Cloning of PHP and HPP: All fragments for the cloning of PHP were PCR amplified from the plasmids pmCherryPAL and MS2-p65-HSF1_GFP. MS2-P65-HSF1_GFP was a gift from Feng Zhang (Addgene plasmid # 61423). The full-length insert was cloned into the pEGFP-N1 (Clontech) backbone, which was amplified by inverse PCR using Phusion Flash High-Fidelity PCR Master Mix. The insert MPH from MS2-P65-HSF1_GFP was cloned into the pEGFP-N1 backbone. For the cloning, Gibson Assembly® Master Mix (NEB, Ipswich) was used according to the manufacturers protocol. HPP was generated from PHP by PCR and AQUA cloning.

dCas9 constructions: As PCR template for the final pdCas9_eGFP, the plasmid dCAS9-VP64_GFP was used. dCAS9-VP64_GFP was a gift from Feng Zhang (Addgene plasmid # 61422; http://n2t.net/addgene:61422; RRID: Addgene_61422). The backbone was amplified by PCR, excluding the VP64 sequence. For the plasmid amplification, Phusion Flash High-Fidelity PCR Master Mix was used. The primers for the insert were designed to have an overlap with the target backbone. The amplicon was phosphorylated and ligated to gain pdCas9_T2A_eGFP.

sgRNA constructions: The sgRNAs containing PAL aptamers were designed using the sgRNA 2.0 scaffold as a template. The stem regions of the tetraloop and stem-loop 2 were preserved and artificial stem sequences were added. The sequences were tested for correct folding using mFold software. As host plasmid pENTR.hU6 was used. The plasmid backbone was amplified using Phusion Flash High-Fidelity PCR Master Mix. The sgRNA inserts were ordered from Ella Biotech (Martinsried, Germany) and cloned into the pENTR.hU6 backbone using AQUA cloning (Table S2, Supporting Information). New seed sequences were introduced by PCR, where the forward primer contains the new seed sequence as 5' extension.

Optogenetic gene activation assays: HeLa cells were seeded at 70.000 cells per well in 24-well plates and cultured for 24h at 37 °C and 5% CO_2. HeLa cells were transfected using Lipofectamine 2000. For the experiments four plasmids were mixed in a specific ratio and a final amount of 200 fmol. The ratio used was 2:1:1:1 (dCas9:Effector:sgRNA:Reporter). For MS2 dependent activation MPH was used. For PAL dependent activation the plasmid PHP or HPP was used. For all experiments the pdCas9-T2A-eGFP plasmid was used for dCas9 expression. For the expression of the sgRNAs, pENTR plasmids that contains a hU6 promoter were used. The transfection was incubated for 4 hours at 37ºC and 5% CO_2 in the presence of blue light of 465 nm or in the absence of light. After the replacement of medium the cells were incubated for 24 hours in the light or in the dark. For flow cytometry analysis, the cells were transfected with the pGL3-Basic-8xgRNA-eBFP reporter plasmid. In short, cells were first washed with 500 µl warm DPBS (Gibco) and then detached using 200 µl Accutase (Gibco). The volume was filled up to 500 µl using cultivation medium and the resuspended cells were transferred into FACS tubes (BD). The cells were centrifuged for 5 min at 200 g. Then the supernatant was removed and the cells were washed with 1 ml warm DPBS. The cells were measured using a BD FACS Canto II. The voltage for FSC was set to 50 V, SSC to 300 V and Pacific Blue Laser (405 nm) to 187 V. The FSC threshold was set to 5.000. HeLa cells were gated for single cells with an auto fluorescence of 1% (Figure S2e, Supporting Information). The activation was normalized using the feature scaling method. For *Metridia* luciferase reporter assays, 50.000 HeLa cells were seeded. As reporter plasmid pGL3-Basic-8xgRNA-metLuc was used. For

measurement, 50 µl of supernatant was collected from each sample and transferred to a LUMITRAC 200 96-well plate. Luciferase reagents were prepared according to the manufacturers manual (Clontech). For the reversible *Metridia* luciferase reporter assays, after transfection 50 µl of supernatant was collected for measurement while the rest was replaced by fresh cell culture medium. The replacement of supernatant was done before every switching illumination setting from light to dark or dark to light.

Optogenetic activation of endogenous ASCL1: HeLa cells were seeded at 70.000 cells per well in 24-well plates and cultured for 24h at 37 °C and 5% CO_2. HeLa cells were transfected using Lipofectamine 2000. For the experiments three plasmids were mixed in a specific ratio and a final amount of 200 fmol. The ratio used was 2:1:1 (dCas9:Effector:sgRNA). For MS2 dependent activation MPH was used. For PAL dependent activation the plasmid PHP was used. For all experiments the pdCas9-T2A-eGFP plasmid was used for dCas9 expression. For the expression of the sgRNAs, pENTR plasmids that contains a hU6 promoter were used. The transfection was incubated for 4 hours at 37ºC and 5% CO_2 in the presence of blue light of 465 nm or in the absence of light. After the replacement of medium the cells were incubated for 24 hours in the light or in the dark. For qPCR analysis, cellular RNA isolation and reverse-transcription were done with the Cells-to-Ct kit (Thermo Fisher Scientific) using TaqMan gene expression master mix (Thermo Fisher Scientific). TaqMan probes were used for ASCL1 and GAPDH detection (Life Technologies; TaqMan gene expression assay IDs were Hs04187546_g1 (ASCL1) and Hs99999905_m1 (GAPDH)). The fold induction of mRNA was calculated by first calculating the Δc_t value for each biological sample. The Δc_t values were then normalized to control sgRNA to obtain $\Delta\Delta c_t$ values. For the fold induction of mRNA, $2^\wedge\Delta\Delta c_t$ was calculated.

Cas9 cleavage assays: First, a 1 µM Cas9 solution was prepared from a 20 µM Cas9 stock solution (NEB, Ipswich) in 1x Cas9 buffer (NEB, Ipswich). The DNA cleavage substrate was amplified from pGL3-Basic-8xgRNA-eBFP. Per assay 150 ng of substrate was used. In the assay molar ratios of 8:1 (Cas9:substrate) and 1:1 (Cas9:gRNA) were used in a final volume of 20 µl. First ddH2O, Cas9 buffer and sgRNA were mixed in a single reaction tube. For sgRNA refolding, the mixture was heated for 2 min at 95 °C and then cooled down to 20 °C with 6 °C/min. For complex formation Cas9 was added and incubated for 5 min at 20 °C. The substrate was added to start the reaction. The cleavage reaction was incubated for 35 min at 37 °C. Subsequently 1 µl of 20 mg/ml Proteinase K (Roth) was added and incubated for 35 min at 55 °C to stop the reaction. The reaction was loaded on a 1% agarose gel. The samples were run for 30 min at 130 V and then stained with ethidium bromide. The cleavage products were visualized using UV irradiation and images were made. The cleavage fractions were analyzed using ImageJ 1.50i software.

In vitro transcription of gRNAs: sgRNA templates with a 5’ attached T7 promoter were ordered from Ella Biotech (Martinsried, Germany). The in vitro transcription was done overnight and the RNA was isolated and purified by PAGE and electro elution.

Statistics: Statistical analysis for pairwise comparisons between light/dark experiments was performed using Welch's two-tailed *t*-test and confidence intervals of 95% in Prism 6.

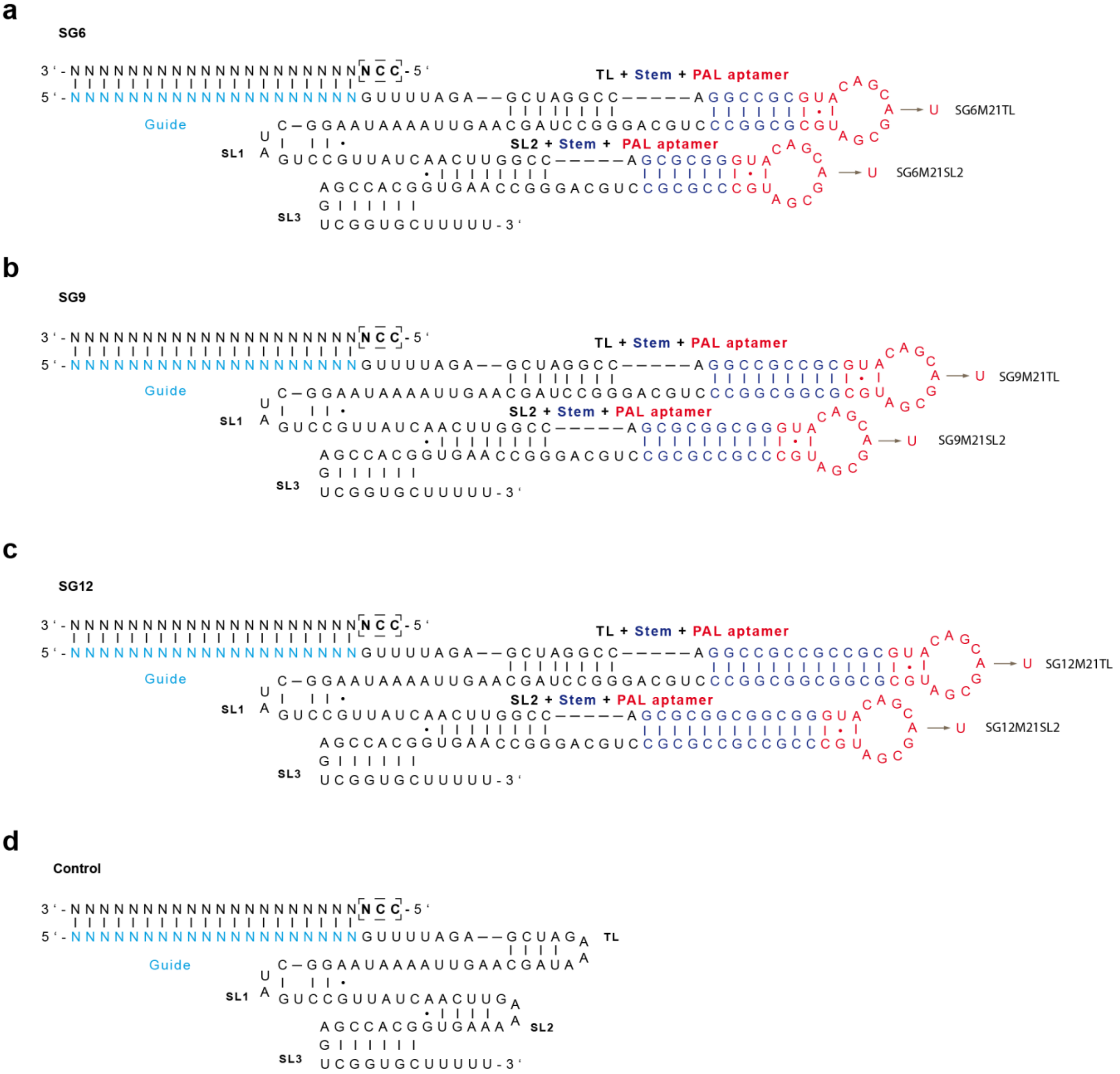

Figure S1. Engineering of sgRNAs. The sgRNAs were extended by inserting the PAL aptamer 53.19 into the TL and SL2. Exchanging the adenine in the aptamer loop region to a uridine leads to the non-binding M21 mutation. (a) sgRNA SG6 with a stem size of 6 nt. (b) sgRNA SG9 with a stem size of 9 nt. (c) sgRNA SG12 with a stem size of 12 nt. (d) control sgRNA without aptamer modification.

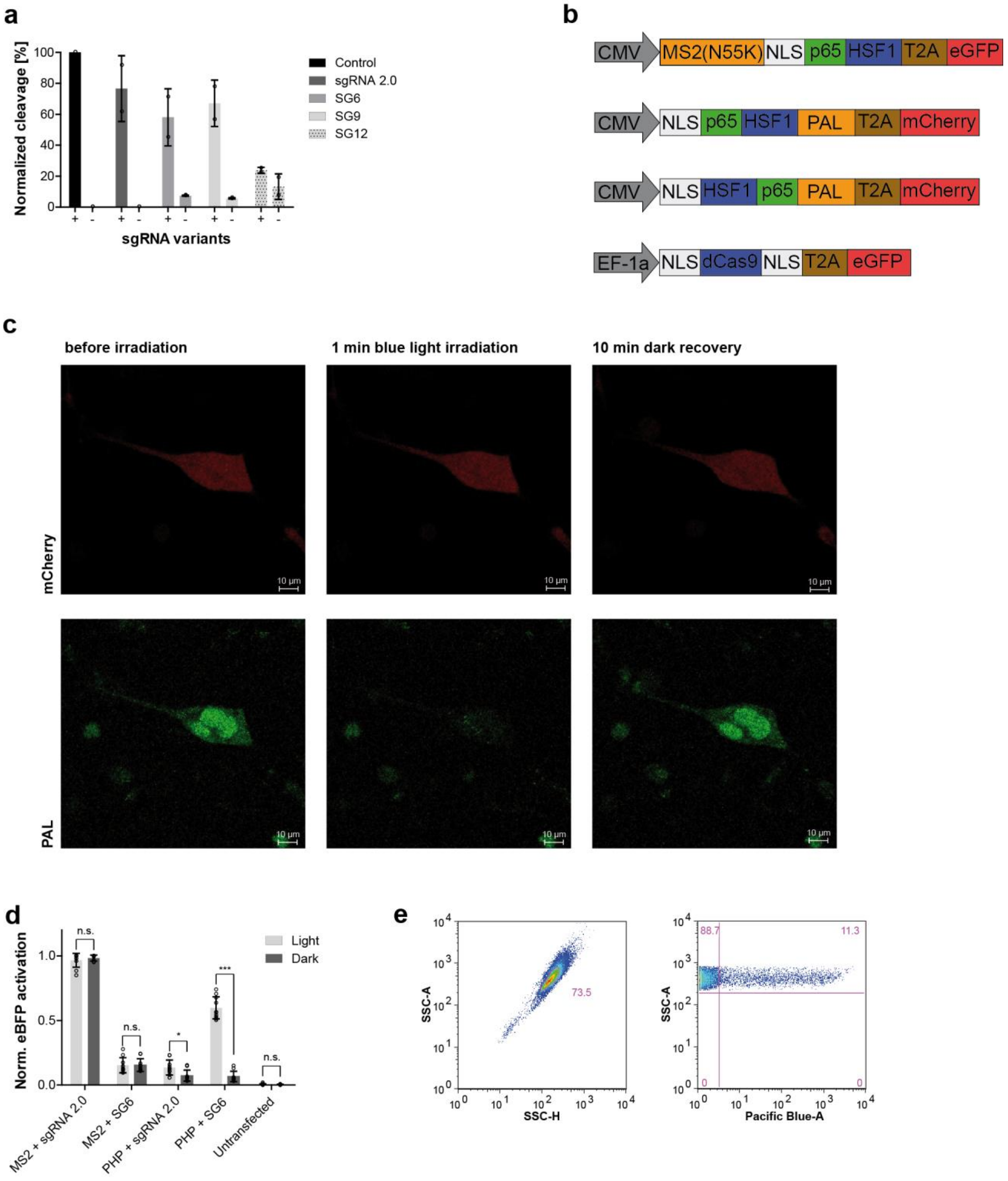
a
Normalized cleavage [%]
100
80
60
40
20
0
sgRNA variants
Control
sgRNA 2.0
SG6
SG9
SG12
b
CMV
MS2(N55K)
NLS
p65
HSF1
T2A
eGFP
PAL
mCherry
EF-1a
dCas9
c
before irradiation
1 min blue light irradiation
10 min dark recovery
mCherry
PAL
10 µm
d
Norm. eBFP activation
1.0
0.5
0.0
n.s.
*
***
Light
Dark
MS2 + sgRNA 2.0
MS2 + SG6
PHP + sgRNA 2.0
PHP + SG6
Untransfected
e
SSC-A
SSC-H
73.5
88.7
11.3
Pacific Blue-A

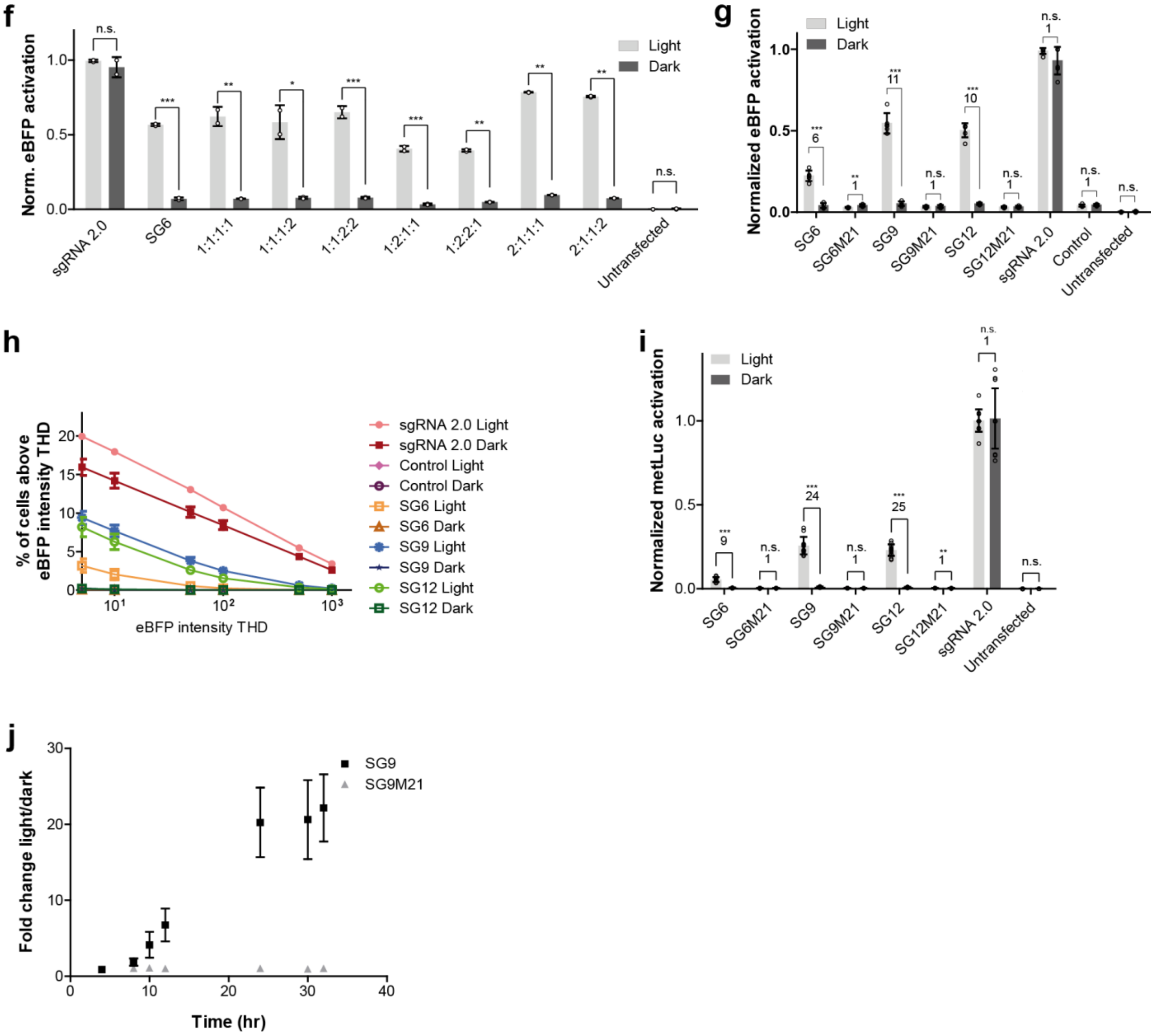

Figure S2. (**a**) Cas9 cleavage assay. The PAL-aptamer modified sgRNAs were tested in a Cas9 cleavage assay. New sgRNAs were designed incorporating the PAL-aptamer with varying stem sizes (SG6, SG9 and SG12) in the TL and SL2 accordingly to the design of sgRNA 2.0. The data is presented as mean values ± s.d and was normalized to the control sgRNA using min-max scaling (n = 2 from 2 individual experiments). (**b**) Expression cassettes of the activating fusion proteins and the dCas9 plasmid. (**c**) Switching activity of the photoreceptor fusion protein PHP. HeLa cells were transfected with PHP and analyzed regarding PAL localization in the nucleus and ubiquitous mCherry expression. The cells were irradiated with blue light to change the conformation of PAL, which leads to a loss of PAL fluorescence. After 10 min recovery in the dark, PAL switches back to the fluorescent dark conformation indicating that the fusion protein can undergo a complete photo cycle. Scale bar = 10 µm. (**d**) Light-induced activation of eBFP expression. HeLa cells were transfected with dCas9, MPH or PHP, eBFP reporter and sgRNA targeting the 8x sgRNA binding sites upstream of the eBFP promoter. The data is presented as mean values ± s.d and was normalized to sgRNA 2.0 control using min-max scaling (n = 10 from 5 individual experiments with two cell culture replicates). Welch's two-tailed *t*-test was performed for the fold induction in the light versus the sample in the dark. The resulting *p*-values of the samples are given in the Supporting Information in Table S3. *p <0.05, **p <0.01, ***p<0.005 versus the sample in the dark. (**e**) Gating strategy for optogenetic eBFP activation assays. HeLa cells were analyzed by flow cytometry and 30.000 events were recorded. From all events, first the main single-cell population was isolated using SSC-A to SSC-H plot. The isolated population was then plotted to visualize the signal intensity of the Pacific Blue-A channel. A quadrant gate was applied, which separates the population from cells with more (Q2) and less (Q1) than 1% fluorescence. Events that have a Pacific Blue-A intensity, which exceeds 1% (Q2 gate) are considered as activated cells. Q2 gate values were exported as table and the values were further processed in Microsoft Excel for normalization. (**f**) Optimization of plasmid ratios for light-induced activation of eBFP expression. HeLa cells were transfected with different ratios of dCas9, MPH or PHP, eBFP reporter and sgRNA targeting the 8x sgRNA binding sites upstream of the eBFP promoter. The data is presented as mean values ± s.d and was normalized to the sgRNA 2.0 control using min-max scaling (n = 6 from 3 individual experiments with two cell culture replicates. Welch's two-tailed *t*-test was performed for the fold induction in the light versus the sample in the dark. The resulting *p*-values of the samples are given in the Supporting Information in Table S3. *p <0.05, **p <0.01, ***p<0.005 versus the sample in the dark. (**g**) Light-dependent upregulation of eBFP using sgRNAs with mutants and varying aptamer stem-lengths showing normalized data. The data is presented as mean values ± s.d and was normalized to the sgRNA 2.0 control

using min-max scaling (n = 6 from 3 individual experiments with two cell culture replicates. Welch's two-tailed *t*-test was performed for the fold induction in the light versus the sample in the dark. The resulting *p*-values of the samples are given in the Supporting Information in Table S3. *p <0.05, **p <0.01, ***p<0.005 versus the sample in the dark. (**h**) Light-dependent upregulation of eBFP using sgRNAs with mutants and varying aptamer stem-lengths. The number of eBFP positive cells gained from **(g)** is presented as a function of eBFP intensity threshold (THD). The data is presented as mean values ± s.d (n = 6 from 3 individual experiments with two cell culture replicates). (**i**) Light-dependent upregulation of *Metridia* Luciferase using sgRNAs with varying aptamer stem-lengths and point mutants showing normalized data. The data is presented as mean values ± s.d and was normalized to the sgRNA 2.0 control using min-max scaling (n = 6 from 3 individual experiments with two cell culture replicates. Welch's two-tailed *t*-test was performed for the fold induction in the light versus the sample in the dark. The resulting *p*-values of the samples are given in the Supporting Information in Table S3. *p <0.05, **p <0.01, ***p<0.005 versus the sample in the dark. (**j**) Light-dependent activation of *Metridia* luciferase expression over time. The data is presented as mean values ± s.d (n = 6 from 3 individual experiments with two cell culture replicates). Welch's two-tailed *t*-test was performed for the fold induction in the light versus the sample in the dark. The resulting *p*-values of the samples are given in the Supporting Information in Table S3. *p <0.05, **p <0.01, ***p<0.005 versus the sample in the dark.

Table S1. Target sequences of sgRNAs.

sgRNA name	Target	Seed sequence 5'->3'
CMV	8x gRNA repeat upstream of minimal CMV promoter	aaaggtcgagaaactgcaaa
Nonsense	No target in human genome	gaacagtcgcgtttgcgact
sgASCL1-1	ASCL1	ggctgggtgtcccattgaaa
sgASCL1-2	ASCL1	gcagccgctcgctgcagcag
sgASCL1-3	ASCL1	gtgtttattcagccgggagtc
sgASCL1-4	ASCL1	gatggagagtttgcaaggagc
sgASCL1-5	ASCL1	gcaggagggacaaatggctct
sgASCL1-6	ASCL1	gagtgaggagagagagaaaac

Table S2. Oligonucleotide sequences for constructing sgRNAs

See attached excel file.

Table S3. Statistical analysis

Figure	Dataset 1	Dataset 2	*p*-value	Significance
2d	HPP + SG9, Dark	HPP + SG9, Light	0.0004	***
	HPP + SG9M21, Dark	HPP + SG9M21, Light	0.1891	n.s.
	PHP + SG9, Dark	PHP + SG9, Light	0.000004	***
	PHP + SG9M21, Dark	PHP + SG9M21, Light	0.1128	n.s.
2e	SG9, Dark	SG9, Light	0.0000002	***
	SG9M21 TL+SL2, Dark	SG9M21 TL+SL2, Light	0.0147	*
	SG9M21 TL, Dark	SG9M21 TL, Light	0.0015	**
	SG9M21 SL2, Dark	SG9M21 SL2, Light	0.0073	**
2g	SG9 12h, Dark	SG9 12h, Light	0.0000003	***
	SG9M21 12h, Dark	SG9M21 12h, Light	0.9621	n.s.
	SG9 24h, Dark	SG9 24h, Light	0.000000005	***
	SG9M21 24h, Dark	SG9M21 24h, Light	0.0092	**
	SG9 36h, Dark	SG9 36h, Light	0.000003	***
	SG9M21 36h, Dark	SG9M21 36h, Light	0.1371	n.s.
3c	sgRNA 2.0, Dark	sgRNA 2.0, Light	0.1191	n.s.
	SG9#2, Dark	SG9#2, Light	0.0003	***
	SG9#4, Dark	SG9#4, Light	0.000007	***
	SG9#2 + SG9#4, Dark	SG9#2 + SG9#4, Light	0.000001	***
S2d	MS2 + sgRNA 2.0, Dark	MS2 + sgRNA 2.0, Light	0.3923	n.s.
	MS2 + SG6, Dark	MS2 + SG6, Light	0.9491	n.s.
	PHP + sgRNA 2.0, Dark	PHP + sgRNA 2.0, Light	0.0133	*
	PHP + SG6, Dark	PHP + SG6, Light	0.0000000006	***
S2f	sgRNA 2.0, Dark	sgRNA 2.0, Light	0.8485	n.s.
	SG6, Dark	SG6, Light	0.0005	***
	1:1:1:1, Dark	1:1:1:1, Light	0.0088	**
	1:1:1:2, Dark	1:1:1:2, Light	0.0175	*

	1:1:2:2, Dark	1:1:2:2, Light	0.0007	***
	1:2:1:1, Dark	1:2:1:1, Light	0.0005	***
	1:2:2:1, Dark	1:2:2:1, Light	0.0074	**
	2:1:1:1, Dark	2:1:1:1, Light	0.001	**
	2:1:1:2, Dark	2:1:1:2, Light	0.0016	**
S2g	SG6, Dark	SG6, Light	0.000004	***
	SG6M21, Dark	SG6M21, Light	0.0059	**
	SG9, Dark	SG9, Light	0.000008	***
	SG9M21, Dark	SG9M21, Light	0.4794	n.s.
	SG12, Dark	SG12, Light	0.000002	***
	SG12M21, Dark	SG12M21, Light	0.5292	n.s.
	sgRNA 2.0, Dark	sgRNA 2.0, Light	0.3876	n.s.
	Control, Dark	Control, Light	0.3251	n.s.
	Untransfected, Dark	Untransfected, Light	0.1485	n.s.
S2i	SG6, Dark	SG6, Light	0.0000001	***
	SG6M21, Dark	SG6M21, Light	0.6822	n.s.
	SG9, Dark	SG9, Light	0.000000005	***
	SG9M21, Dark	SG9M21, Light	0.0844	n.s.
	SG12, Dark	SG12, Light	0.0000000002	***
	SG12M21, Dark	SG12M21, Light	0.0031	**
	sgRNA 2.0, Dark	sgRNA 2.0, Light	0.8254	n.s.
	Untransfected, Dark	Untransfected, Light	0.9565	n.s.
S2j	SG9 4h, Dark	SG9 4h, Light	0.0752	n.s.
	SG9 8h, Dark	SG9 8h, Light	0.0031	**
	SG9 10h, Dark	SG9 10h, Light	0.0002	***
	SG9 12h, Dark	SG9 12h, Light	0.00000004	***
	SG9 24h, Dark	SG9 24h, Light	0.000003	***
	SG9 30h, Dark	SG9 30h, Light	0.0000004	***
	SG9 32h, Dark	SG9 32h, Light	0.00000002	***
	SG9M21 4h, Dark	SG9M21 4h, Light	0.1234	n.s.
	SG9M21 8h, Dark	SG9M21 8h, Light	0.9146	n.s.
	SG9M21 10h, Dark	SG9M21 10h, Light	0.7348	n.s.
	SG9M21 12h, Dark	SG9M21 12h, Light	0.6816	n.s.
	SG9M21 24h, Dark	SG9M21 24h, Light	0.5432	n.s.
	SG9M21 30h, Dark	SG9M21 30h, Light	0.7029	n.s.
	SG9M21 32h, Dark	SG9M21 32h, Light	0.9363	n.s.

Note S1. Construction of modified sgRNA sequences

Color code:
gRNA Backbone
Stemregion for aptamer
aptamer

Control sgRNA
NNNNNNNNNNNNNNNNNNNNGTTTTAGAGCTAGAAATAGCAAGTTAAAATAAGGCTAGTCCGTTATCAACTTGAAAAAGTGGCACCGAGT
CGGTGCTTTTT

sgRNA 2.0
NNNNNNNNNNNNNNNNNNNNGTTTTAGAGCTA
GGCC AACATGAGGATCACCCATGTCTGCAG GGCC
TAGCAAGTTAAAATAAGGCTAGTCCGTTATCAACTT
GGCC AACATGAGGATCACCCATGTCTGCAG GGCC
AAGTGGCACCGAGTCGGTGCTTTTT

SG6
NNNNNNNNNNNNNNNNNNNNGTTTTAGAGCTAGGCCA
GGCCGC GTACAGCAGCGATGC GCGGCC
CTGCAG GGCCTAGCAAGTTAAAATAAGGCTAGTCCGTTATCAACTT GGCCA
GCGCGG GTACAGCAGCGATGC CCGCGC
CTGCAGGGCCAAGTGGCACCGAGTCGGTGCTTTTT

SG6M21
NNNNNNNNNNNNNNNNNNNNGTTTTAGAGCTAGGCCA
GGCCGC GTACAGCTGCGATGC GCGGCC
CTGCAG GGCCTAGCAAGTTAAAATAAGGCTAGTCCGTTATCAACTT GGCCA
GCGCGG GTACAGCTGCGATGC CCGCGC
CTGCAGGGCCAAGTGGCACCGAGTCGGTGCTTTTT

SG9
NNNNNNNNNNNNNNNNNNNNGTTTTAGAGCTAGGCCA
GGCCGCCGC GTACAGCAGCGATGC GCGGCGGCC

CTGCAG GGCCTAGCAAGTTAAAATAAGGCTAGTCCGTTATCAACTT GGCCA
GCGCGGCGG GTACAGCAGCGATGC CCGCCGCGC
CTGCAGGGCCAAGTGGCACCGAGTCGGTGCTTTTT

SG9M21
NNNNNNNNNNNNNNNNNNNNGTTTTAGAGCTAGGCCA
GGCCGCCGC GTACAGCTGCGATGC GCGGCGGCC
CTGCAG GGCCTAGCAAGTTAAAATAAGGCTAGTCCGTTATCAACTT GGCCA
GCGCGGCGG GTACAGCTGCGATGC CCGCCGCGC
CTGCAGGGCCAAGTGGCACCGAGTCGGTGCTTTTT

SG9M21TL
NNNNNNNNNNNNNNNNNNNNGTTTTAGAGCTAGGCCA
GGCCGCCGC GTACAGCTGCGATGC GCGGCGGCC
CTGCAG GGCCTAGCAAGTTAAAATAAGGCTAGTCCGTTATCAACTT GGCCA
GCGCGGCGG GTACAGCAGCGATGC CCGCCGCGC
CTGCAGGGCCAAGTGGCACCGAGTCGGTGCTTTTT

SG9M21SL2
NNNNNNNNNNNNNNNNNNNNGTTTTAGAGCTAGGCCA
GGCCGCCGC GTACAGCAGCGATGC GCGGCGGCC
CTGCAG GGCCTAGCAAGTTAAAATAAGGCTAGTCCGTTATCAACTT GGCCA
GCGCGGCGG GTACAGCTGCGATGC CCGCCGCGC
CTGCAGGGCCAAGTGGCACCGAGTCGGTGCTTTTT

SG12
NNNNNNNNNNNNNNNNNNNNGTTTTAGAGCTAGGCCA
GGCCGCCGCCGC GTACAGCAGCGATGC GCGGCGGCGGCC
CTGCAG GGCCTAGCAAGTTAAAATAAGGCTAGTCCGTTATCAACTT GGCCA
GCGCGGCGGCGG GTACAGCAGCGATGC CCGCCGCCGCGC
CTGCAGGGCCAAGTGGCACCGAGTCGGTGCTTTTT

SG12M21
NNNNNNNNNNNNNNNNNNNNGTTTTAGAGCTAGGCCA
GGCCGCCGCCGC GTACAGCTGCGATGC GCGGCGGCGGCC
CTGCAG GGCCTAGCAAGTTAAAATAAGGCTAGTCCGTTATCAACTT GGCCA
GCGCGGCGGCGG GTACAGCTGCGATGC CCGCCGCCGCGC
CTGCAGGGCCAAGTGGCACCGAGTCGGTGCTTTTT

Note S2. Amino acid sequences of fusion proteins

MS2(N55K)-p65-HSF1-T2A-eGFP
MASNFTQFVLVDNGGTGDVTVAPSNFANGVAEWISSNSRSQAYKVTCSVRQSSAQKRKYTIKVEVPKVATQTVGGVELPVAAWRSYLNMELTI
PIFATNSDCELIVKAMQGLLKDGNPIPSAIAANSGIYSAGGGGSGGGGSGGGGSGPKKKRKVAAAGSPSGQISNQALALAPSSAPVLAQTMVP
SSAMVPLAQPPAPAPVLTPGPPQSLSAPVPKSTQAGEGTLSEALLHLQFDADEDLGALLGNSTDPGVFTDLASVDNSEFQQLLNQGVSMSHST
AEPMLMEYPEAITRLVTGSQRPPDPAPTPLGTSGLPNGLSGDEDFSSIADMDFSALLSQISSSGQGGGGSGFSVDTSALLDLFSPSVTVPDMSL
PDLDSSLASIQELLSPQEPPRPPEAENSSPDSGKQLVHYTAQPLFLLDPGSVDTGSNDLPVLFELGEGSYFSEGDGFAEDPTISLLTGSEPPKAK
DPTVSASGSGEGRGSLLTCGDVEENPGPVSKGEELFTGVVPILVELDGDVNGHKFSVSGEGEGDATYGKLTLKFICTTGKLPVPWPTLVTTLTY
GVQCFSRYPDHMKQHDFFKSAMPEGYVQERTIFFKDDGNYKTRAEVKFEGDTLVNRIELKGIDFKEDGNILGHKLEYNYNSHNVYIMADKQKN
GIKVNFKIRHNIEDGSVQLADHYQQNTPIGDGPVLLPDNHYLSTQSALSKDPNEKRDHMVLLEFVTAAGITLGMDELYK-

NLS-p65-HSF1-PAL-T2A-mCherry
MPKKKRKVAAAGSPSGQISNQALALAPSSAPVLAQTMVPSSAMVPLAQPPAPAPVLTPGPPQSLSAPVPKSTQAGEGTLSEALLHLQFDADED
LGALLGNSTDPGVFTDLASVDNSEFQQLLNQGVSMSHSTAEPMLMEYPEAITRLVTGSQRPPDPAPTPLGTSGLPNGLSGDEDFSSIADMDFS
ALLSQISSSGQGGGGSGGCTTCAGCGTGGACACCAGTGCCCTGCTGGACCTGTTCAGCCCCTCGGTGACCGTGCCCGACATGAGCCTGC
CTGACCTTGACAGCAGCCTGGCCAGTATCCAAGAGCTCCTGTCTCCCCAGGAGCCCCCCAGGCCTCCCGAGGCAGAGAACAGCAGCCC
GGATTCAGGGAAGCAGCTGGTGCACTACACAGCGCAGCCGCTGTTCCTGCTGGACCCCGGCTCCGTGGACACCGGGAGCAACGACCT
GCCGGTGCTGTTTGAGCTGGGAGAGGGCTCCTACTTCTCCGAAGGGGACGGCTTCGCCGAGGACCCCACCATCTCCCTGCTGACAGGC
TCGGAGCCTCCCAAAGCCAAGGACCCCACTGTCTCCGGSGGGSGGGSGKVNRPAERASFGSFVLDAGSARFVGSDELALVLGFAPGDVVL
TPAVVLAHLHPDDRLEWQAGLQRCLATGRPVVVNHLLLTAEAEPRPAMTTLTALTEQDRVRAVTGVITDLSDRVRRATEAEIRQAVRAAAATRS
EIDQAKGIVMAAFDVDADQAFALLKWHSSQSNRKLRDLATGMIEGLAAANSALPLRRRLSTVFTDMGCPAPSTKGWTVPVTDIGLPPTSGLIPT
ALLPGILTRAAHDASVAITVADVTAPDQPLVYANPAFERLTGYAAAEVLGRNCRFLQAESGDPHERSAIRSAIANGDAVTTLIRNFRQDGHAFWN
EFHLSPVRNGAGRVTHYIGYQLDVTERVERDQQLEQLASEGRGSLLTCGDVEENPGPMVSKGEEDNMAIIKEFMRFKVHMEGSVNGHEFEIE
GEGEGRPYEGTQTAKLKVTKGGPLPFAWDILSPQFMYGSKAYVKHPADIPDYLKLSFPEGFKWERVMNFEDGGVVTVTQDSSLQDGEFIYKVK
LRGTNFPSDGPVMQKKTMGWEASSERMYPEDGALKGEIKQRLKLKDGGHYDAEVKTTYKAKKPVQLPGAYNVNIKLDITSHNEDYTIVEQYER
AEGRHSTGGMDELYK-

NLS-HSF1-p65-PAL-T2A-mCherry
MPKKKRKVSGQGGGGSGFSVDTSALLDLFSPSVTVPDMSLPDLDSSLASIQELLSPQEPPRPPEAENSSPDSGKQLVHYTAQPLFLLDPGSVD
TGSNDLPVLFELGEGSYFSEGDGFAEDPTISLLTGSEPPKAKDPTVSAAAGSPSGQISNQALALAPSSAPVLAQTMVPSSAMVPLAQPPAPAPV
LTPGPPQSLSAPVPKSTQAGEGTLSEALLHLQFDADEDLGALLGNSTDPGVFTDLASVDNSEFQQLLNQGVSMSHSTAEPMLMEYPEAITRLV
TGSQRPPDPAPTPLGTSGLPNGLSGDEDFSSIADMDFSALLSQISSGGSGGGSGGGSGKVNRPAERASFGSFVLDAGSARFVGSDELALVLG
FAPGDVVLTPAVVLAHLHPDDRLEWQAGLQRCLATGRPVVVNHLLLTAEAEPRPAMTTLTALTEQDRVRAVTGVITDLSDRVRRATEAEIRQAV
RAAAATRSEIDQAKGIVMAAFDVDADQAFALLKWHSSQSNRKLRDLATGMIEGLAAANSALPLRRRLSTVFTDMGCPAPSTKGWTVPVTDIGLP
PTSGLIPTALLPGILTRAAHDASVAITVADVTAPDQPLVYANPAFERLTGYAAAEVLGRNCRFLQAESGDPHERSAIRSAIANGDAVTTLIRNFRQ
DGHAFWNEFHLSPVRNGAGRVTHYIGYQLDVTERVERDQQLEQLASEGRGSLLTCGDVEENPGPMVSKGEEDNMAIIKEFMRFKVHMEGSV
NGHEFEIEGEGEGRPYEGTQTAKLKVTKGGPLPFAWDILSPQFMYGSKAYVKHPADIPDYLKLSFPEGFKWERVMNFEDGGVVTVTQDSSLQ
DGEFIYKVKLRGTNFPSDGPVMQKKTMGWEASSERMYPEDGALKGEIKQRLKLKDGGHYDAEVKTTYKAKKPVQLPGAYNVNIKLDITSHNED
YTIVEQYERAEGRHSTGGMDELYK-

NLS-dCas9(D10A, H840A)-NLS-T2A-eGFP
MSPKKKRKVEASDKKYSIGLAIGTNSVGWAVITDEYKVPSKKFKVLGNTDRHSIKKNLIGALLFDSGETAEATRLKRTARRRYTRRKNRICYLQEI
FSNEMAKVDDSFFHRLEESFLVEEDKKHERHPIFGNIVDEVAYHEKYPTIYHLRKKLVDSTDKADLRLIYLALAHMIKFRGHFLIEGDLNPDNSDV
DKLFIQLVQTYNQLFEENPINASGVDAKAILSARLSKSRRLENLIAQLPGEKKNGLFGNLIALSLGLTPNFKSNFDLAEDAKLQLSKDTYDDDLDNL
LAQIGDQYADLFLAAKNLSDAILLSDILRVNTEITKAPLSASMIKRYDEHHQDLTLLKALVRQQLPEKYKEIFFDQSKNGYAGYIDGGASQEEFYKF
IKPILEKMDGTEELLVKLNREDLLRKQRTFDNGSIPHQIHLGELHAILRRQEDFYPFLKDNREKIEKILTFRIPYYVGPLARGNSRFAWMTRKSEETI
TPWNFEEVVDKGASAQSFIERMTNFDKNLPNEKVLPKHSLLYEYFTVYNELTKVKYVTEGMRKPAFLSGEQKKAIVDLLFKTNRKVTVKQLKED
YFKKIECFDSVEISGVEDRFNASLGTYHDLLKIIKDKDFLDNEENEDILEDIVLTLTLFEDREMIEERLKTYAHLFDDKVMKQLKRRRYTGWGRLSR
KLINGIRDKQSGKTILDFLKSDGFANRNFMQLIHDDSLTFKEDIQKAQVSGQGDSLHEHIANLAGSPAIKKGILQTVKVVDELVKVMGRHKPENIVI
EMARENQTTQKGQKNSRERMKRIEEGIKELGSQILKEHPVENTQLQNEKLYLYYLQNGRDMYVDQELDINRLSDYDVDAIVPQSFLKDDSIDNK
VLTRSDKNRGKSDNVPSEEVVKKMKNYWRQLLNAKLITQRKFDNLTKAERGGLSELDKAGFIKRQLVETRQITKHVAQILDSRMNTKYDENDKL
IREVKVITLKSKLVSDFRKDFQFYKVREINNYHHAHDAYLNAVVGTALIKKYPKLESEFVYGDYKVYDVRKMIAKSEQEIGKATAKYFFYSNIMNFF
KTEITLANGEIRKRPLIETNGETGEIVWDKGRDFATVRKVLSMPQVNIVKKTEVQTGGFSKESILPKRNSDKLIARKKDWDPKKYGGFDSPTVAY
SVLVVAKVEKGKSKKLKSVKELLGITIMERSSFEKNPIDFLEAKGYKEVKKDLIIKLPKYSLFELENGRKRMLASAGELQKGNELALPSKYVNFLYL
ASHYEKLKGSPEDNEQKQLFVEQHKHYLDEIIEQISEFSKRVILADANLDKVLSAYNKHRDKPIREQAENIIHLFTLTNLGAPAAFKYFDTTIDRKR
YTSTKEVLDATLIHQSITGLYETRIDLSQLGGDSAGGGGSGGGGSGGGGSGPKKKRKVAAINASGSGEGRGSLLTCGDVEENPGPVSKGEELF
TGVVPILVELDGDVNGHKFSVSGEGEGDATYGKLTLKFICTTGKLPVPWPTLVTTLTYGVQCFSRYPDHMKQHDFFKSAMPEGYVQERTIFFKD
DGNYKTRAEVKFEGDTLVNRIELKGIDFKEDGNILGHKLEYNYNSHNVYIMADKQKNGIKVNFKIRHNIEDGSVQLADHYQQNTPIGDGPVLLPD
NHYLSTQSALSKDPNEKRDHMVLLEFVTAAGITLGMDELYK-
